# Supplementary material for: User concerns regarding information sharing on social networking sites: The user’s perspective in the context of national culture
Source: PLoS One. 2022 Jan 31;17(1):e0263157. doi: 10.1371/journal.pone.0263157 (PMC8803144; doi:10.1371/journal.pone.0263157)
Supplement: Attachment — Submitted filename: Response to reviewers.docx [file pone.0263157.s002.docx]

Dr Luigi Lavorgna

Academic Editor

PLOS ONE

5^th^ Jan 2022

Dear Dr Lavorgna,

This letter accompanies resubmission of our paper

PONE-D-21-28070
User concerns regarding information sharing on social networking sites: The user’s perspective

We very much appreciate the work of the Reviewers, which we feel has helped us to improve the paper substantially. We offer responses to their comments as follows.

1. The argument tries to be clear about how it is based on the data. We have tried to clarify the argument in places. We believe that our procedure is rigorous and sound.

2. We believe that our data and analysis are rigorous and have done what we can to check these.

3. Our data are fully available.

4. The manuscript has been substantially written, and thoroughly edited, by an experienced native speaker of English. We believe there are no major errors.

5.
Reviewer #1: The comment is correct, that there is a relationship with another published paper, as noted [full citation: Mutambik I, Almuqrin A, Liu Y, Alhossayin M, Qintash FH. Gender Differentials on Information Sharing and Privacy Concerns on Social Networking Sites. J Glob Inf Manag. 2021 29(3):236–55. DOI 10.4018/JGIM.2021050110], which is closer than was anticipated at the time of submission. We have revised the manuscript (especially on pages 14-20, 27) to make this relationship clear and avoid duplication. The two papers are based on data from the same empirical study, but the focus, analysis and discussion are different. Specifically, the present paper includes in the analysis the construct of Attitude, which we see as important to the argument concerning culture, which does not arise in the other paper. The other paper is focussed on issues of gender: since these are not a central concern in the present paper, we have removed those sections which addressed gender specifically. We feel that this makes the present paper clearer in focus and in structure, as well as a little shorter. The discussion section in the paper is relatively long, but we feel this is necessary to explain how and why our perspective diverges from that which is commonly found in the literature, taking culture into account. We propose a revision of the title of the paper to help emphasise the focus of the paper, to read:
User concerns regarding information sharing on social networking sites: The user’s perspective in the context of national culture.

Reviewer #2: We recognise the importance of the points raised by this Reviewer, and the appropriateness of the additional references suggested, which we have been very happy to include and for which we are grateful. Our focus is on the concerns of academic users sharing information – perhaps they tend to take unduly for granted the accuracy of the information, and should think carefully about how it will be perceived, but these are large issues that would demand a very thorough discussion. In the paper, we have chosen to focus particularly on privacy because this is a particularly salient concern of the users we have studied. We argue there are cultural specificities in the nature of these concerns, but there is much work still to be done in this area and we do not have data of our own from other cultures for direct comparison. We have revised the manuscript to take the Reviewer’s suggestions into account and to try to clarify the focus and limitations of our discussion (mainly pages 3, 6-7, 45).

Sincerely,

John Lee, Ph.D.

School of Informatics

The University of Edinburgh

Edinburgh, UK

+447876196878

[John.Lee@ed.ac.uk](mailto:John.Lee@ed.ac.uk)
